# Integrating GLP-1 receptor agonists with anti-amyloid immunotherapy for Alzheimer’s disease: a phased clinical roadmap

**DOI:** 10.1093/lifemedi/lnaf036

**Published:** 2025-11-14

**Authors:** Junzhe Huang, Shengya Wang, Vincent C T Mok, Weihong Song, Ho Ko, Yun Zhang

**Affiliations:** Gerald Choa Neuroscience Institute, The Chinese University of Hong Kong, Hong Kong SAR 999077, China; Department of Medicine and Therapeutics, Faculty of Medicine, The Chinese University of Hong Kong, Hong Kong SAR 999077, China; Li Ka Shing Institute of Health Sciences, Faculty of Medicine, The Chinese University of Hong Kong, Hong Kong SAR 999077, China; Margaret K.L. Cheung Research Centre for Management of Parkinsonism, Faculty of Medicine, The Chinese University of Hong Kong, Hong Kong SAR 999077, China; Lau Tat-chuen Research Centre of Brain Degenerative Diseases in Chinese, Faculty of Medicine, The Chinese University of Hong Kong, Hong Kong SAR 999077, China; Oujiang Laboratory and Wenzhou Medical University, Wenzhou 325035, China; Gerald Choa Neuroscience Institute, The Chinese University of Hong Kong, Hong Kong SAR 999077, China; Department of Medicine and Therapeutics, Faculty of Medicine, The Chinese University of Hong Kong, Hong Kong SAR 999077, China; Li Ka Shing Institute of Health Sciences, Faculty of Medicine, The Chinese University of Hong Kong, Hong Kong SAR 999077, China; Margaret K.L. Cheung Research Centre for Management of Parkinsonism, Faculty of Medicine, The Chinese University of Hong Kong, Hong Kong SAR 999077, China; Lau Tat-chuen Research Centre of Brain Degenerative Diseases in Chinese, Faculty of Medicine, The Chinese University of Hong Kong, Hong Kong SAR 999077, China; Oujiang Laboratory and Wenzhou Medical University, Wenzhou 325035, China; Gerald Choa Neuroscience Institute, The Chinese University of Hong Kong, Hong Kong SAR 999077, China; Department of Medicine and Therapeutics, Faculty of Medicine, The Chinese University of Hong Kong, Hong Kong SAR 999077, China; Li Ka Shing Institute of Health Sciences, Faculty of Medicine, The Chinese University of Hong Kong, Hong Kong SAR 999077, China; Margaret K.L. Cheung Research Centre for Management of Parkinsonism, Faculty of Medicine, The Chinese University of Hong Kong, Hong Kong SAR 999077, China; Lau Tat-chuen Research Centre of Brain Degenerative Diseases in Chinese, Faculty of Medicine, The Chinese University of Hong Kong, Hong Kong SAR 999077, China; Department of Neurology, Nanjing Drum Tower Hospital, Affiliated Hospital of Medical School, Nanjing University, Nanjing 210000, China

Targeting amyloid β (Aβ) for Alzheimer’s disease (AD) treatment encountered many failures and had historically been a graveyard of such efforts. The setbacks were driven by multiple critical challenges, including the enrolment of substantial numbers of patients misdiagnosed with AD in previous clinical trials, the polymorphic nature of Aβ aggregates creating difficulties for therapeutics to target, and the lack of reliable markers to assess target engagement or dose-response relationships required for therapeutic readouts. The blood–brain barrier poses yet another obstacle, restricting efficient penetration of potential anti-Aβ immunotherapies and other pharmacological agents to the brain parenchyma. Lecanemab, designed to selectively target soluble Aβ protofibrils, and donanemab, which targets N-truncated Aβ in established plaques, have each demonstrated the capacity to effectively drive Aβ plaque clearance in the human brain. Both lecanemab and donanemab activate microglial phagocytosis via Fcγ receptors, with clinical trials reporting modest but meaningful slowing of cognitive decline [1]. These advances provide critical lessons for the development of next-generation AD therapeutics.

Alongside these developments, GLP-1 receptor agonists (GLP-1RAs), originally developed for treating type 2 diabetes mellitus (T2DM) and obesity, both key AD risk factors, have emerged as promising neuroprotective agents. Preclinical studies have shown that GLP-1RAs reduce Aβ and tau pathology, inhibit neuroinflammation, as well as enhance synaptic plasticity and cerebral glucose metabolism. Clinical evidence highlights a negative correlation between plasma GLP-1 levels and Aβ burden in the brains of AD patients [2]. Mechanistically, GLP-1RAs activate AMPK signaling in microglia to promote Aβ phagocytosis [2]. Moreover, GLP-1RAs such as exenatide has demonstrated body-wide anti-aging effects at multi-omic levels rivaling mTOR inhibition, with robust age-counteraction across the transcriptome and DNA methylome of the hippocampus—a key brain region vulnerable in AD [3]. Ongoing Phase III trials, EVOKE and EVOKE+, are directly testing the potential of GLP-1RAs to slow cognitive decline in early symptomatic AD [4].

A dynamic, phased approach may maximize the therapeutic success of both anti-Aβ immunotherapies and GLP-1RAs. AD pathology accumulates silently for up to decades before symptoms appear, creating a critical window for prevention. GLP-1RAs, through their multifaceted effects on metabolism, inflammation, and microglial function, are well positioned as prophylactic agents. In addition, their age-counteracting properties could further bolster brain resilience against early pathological insults [3]. An ongoing preclinical study conducted by us aims to investigate whether restoring homeostasis by a GLP-1RA renders the aging brain more resilient to pathological insults, including Aβ aggregates. Initiating GLP-1RA monotherapy in pre-symptomatic individuals with early AD pathology, regardless of the presence of metabolic risk factors, may slow neuropathological progression. This hypothesis warrants dedicated primary prevention trials, including subjects without diabetes or obesity, to fully define the prophylactic potential of GLP-1RAs. Smart designs, including early readouts for potential pathophysiological modulation and robust measurable endpoints, are required for successful execution of such studies.

The mild cognitive impairment (MCI) stage represents another key therapeutic window, as evidenced by the clinical benefits of anti-Aβ immunotherapies [1]. Mechanistic compatibility between anti-Aβ immunotherapies and GLP-1RAs hinges on their convergent yet complementary actions on microglia. Anti-Aβ immunotherapies such as lecanemab induce a pro-phagocytic microglial phenotype via FcγR engagement, upregulating TREM2, APOE, complement and phagosomal pathways for Aβ clearance [5]. GLP-1RAs enhance Aβ uptake by microglia and suppress inflammatory cascades [2]. Importantly, preclinical validation is required to confirm that GLP-1RA co-administration does not blunt, but rather permits or even enhances, anti-Aβ immunotherapy-mediated microglial Aβ clearance. If confirmed, combining therapy could yield synergy by amplifying microglial phagocytosis, whereby immunotherapies provide targeted opsonization while GLP-1RAs further promote Aβ uptake.

Management strategy after Aβ plaque removal remains poorly defined. Under current practice, donanemab is discontinued upon achieving amyloid negativity, whereas lecanemab is continued with an extended interval regimen and is also offered with an option of home-administered formulation. However, clinical simulations predict Aβ plaque reaccumulating rate of 2.8 centiloids per year after discontinuation of donanemab. More importantly, Aβ clearance alone does not resolve other AD pathologies, such as tauopathy, detrimental neuroinflammation, and metabolic dysfunction, which are key drivers of progressive degeneration. GLP-1RAs may be uniquely suited for long-term maintenance, sustaining brain homeostasis through metabolic and anti-inflammatory effects. Their ability to promote microglial resilience and support synaptic function could be critical for slowing Aβ pathology relapse and preserving cognitive functions. Clinical trials exploring the role of GLP-1RAs as a maintenance therapy post-Aβ-immunotherapy thus represent a logical and necessary next step.

**Figure 1.**
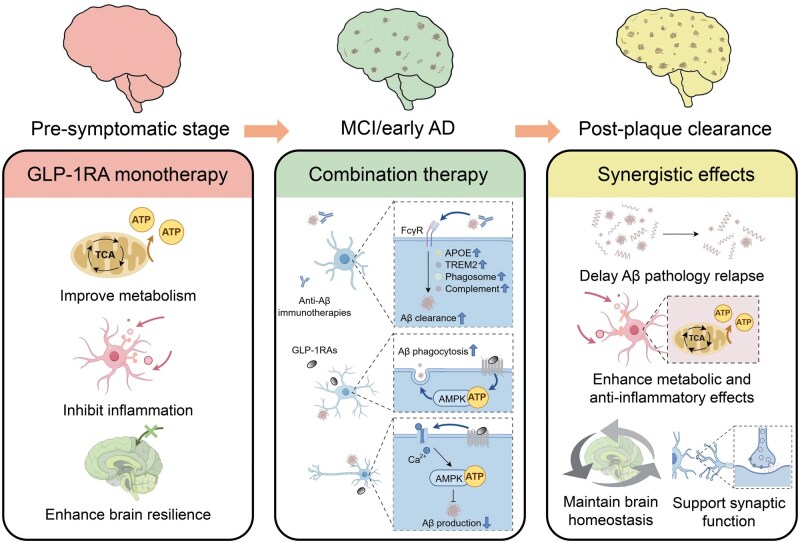
Schematic illustration of GLP-1 receptor agonists (GLP-1RAs) as a therapeutic approach across different stages of Alzheimer’s disease (AD). In the pre-symptomatic stage, GLP-1RA monotherapy improves metabolism, inhibits inflammation, and enhances brain resilience. During mild cognitive impairment or early AD, combination therapy with GLP-1RAs and anti-Aβ immunotherapies enhances amyloid clearance through FcγR, complement, phagosome, APOE, and TREM2 signaling, promotes Aβ phagocytosis, and regulates Aβ production via AMPK-dependent pathways. Following plaque clearance, synergistic effects help delay Aβ pathology relapse, amplify metabolic and anti-inflammatory benefits, maintain brain homeostasis, and preserve synaptic function.

We propose a phased therapeutic roadmap: GLP-1RA monotherapy for pre-symptomatic prophylaxis, combination therapy with anti-Aβ immunotherapies in the early symptomatic stages, and GLP-1RA-driven maintenance after plaque clearance to preserve brain homeostasis (Fig. 1). By leveraging the complementary strengths of targeted Aβ removal and broad neuroprotective actions, this strategy has the potential to revolutionize AD management. We envision this roadmap paving the way for a new paradigm of comprehensive, multi-target therapeutics designed to address the full complexity of AD. Despite compelling rationale, additional preclinical and clinical studies are necessary to validate the efficacy and safety of such a combination therapy approach. Critical questions include whether current GLP-1RA formulations achieve sufficient brain penetration and target engagement in humans, their long-term safety profiles in possible non-diabetic/non-obese populations, the confirmation of stage-specific microglial ­functions throughout the treatment course and their responsiveness to GLP-1RA modulation in the AD brain microenvironment.
